# Effect of Oat Fiber Preparations with Different Contents of β-Glucan on the Formation of Acrylamide in Dietary Bread (Rusks)

**DOI:** 10.3390/molecules29020306

**Published:** 2024-01-07

**Authors:** Karolina Miśkiewicz, Justyna Rosicka-Kaczmarek, Gabriela Kowalska, Agnieszka Maher, Joanna Oracz

**Affiliations:** 1 Institute of Food Technology and Analysis, Faculty of Biotechnology and Food Sciences, Lodz University of Technology, Stefanowskiego Street 2/22, 90-537 Lodz, Poland; karolina.miskiewicz@p.lodz.pl (K.M.); gabriela.kowalska@p.lodz.pl (G.K.); joanna.oracz@p.lodz.pl (J.O.); 2 Department of Environmental Biotechnology, Faculty of Biotechnology and Food Sciences, Lodz University of Technology, Wólczańska Street 171/173, 90-924 Lodz, Poland; agnieszka.maher@dokt.p.lodz.pl

**Keywords:** rusks, acrylamide, oat β-glucans, asparagine, browning index, water activity

## Abstract

In the literature, there are few reports indicating hydrocolloids as a factor capable of reducing the amount of acrylamide formed in food. Therefore, the aim of the study was to examine the ability of soluble oat fiber to reduce the amount of acrylamide formed in the process of obtaining rusks. The effect of the concentration of β-glucans in oat fiber preparations at 20% and 30% and the amount of preparations used at 10%, 15%, and 20% was investigated. On the basis of the obtained test results, it was shown that the most optimal concentration of oat fiber preparation in rusks recipe is at 15%, regardless of the content of β-glucan in it. This concentration makes it possible to reduce the amount of acrylamide formed in baked goods and rusks by ~70% and ~60%, respectively, while maintaining the desired physical and chemical properties of the product. In addition, it was shown that the browning index and water activity strongly correlate with the content of acrylamide in rusks, which makes them good markers of this compound in rusks. The use of hydrocolloids in the form of oat fiber preparations with different contents of β-glucan as a tool for reducing the amount of acrylamide in rusks, at the same time, offers the possibility of enriching these products with a soluble dietary fiber with health properties.

## 1. Introduction

Food health quality is an essential attribute of any food product. This statement is particularly essential in the context of increasing reports regarding health and life risks that are associated, among others, with the presence of acrylamide in heat-treated foods [1,2].

Dietary bread, including rusks, belongs to a group of products intended for children, the elderly, and convalescents. Considering the conditions under which the rusks are baked and dried (i.e., a temperature above 120 °C and a chemical composition of raw materials used for bread making), rusks are potential sources of substances that are of an antinutritional nature, such as acrylamide and its derivatives [3]. The acrylamide content of rusks ranges 30.7–637.0 µg kg^−1^ [4,5]. Acrylamide is synthesized during the Maillard reaction, a process wherein flavor and aroma compounds in thermally processed products are produced. Acrylamide, as a potential human carcinogen, can contribute to the occurrence of many diseases, including those with a genetic background [6]. Therefore, it is necessary to monitor and determine whether it is possible to reduce the acrylamide content in food. Over the last decade, many methods for the reduction of this potential carcinogenic compound in food products have been described. Among them, the following methods can be distinguished: addition of calcium salt [3,7]; replacement of reducing sugars, such as glucose and fructose, with sucrose [8]; enzymatic application of asparaginase prior to thermal processing, employed to diminish the concentrations of asparagine in food products [9,10]; replacement of ammonium bicarbonate with sodium bicarbonate [11,12]; use of a lower pH of the environment during the technological process by adding organic acids (i.e., lactic, tartaric, and citric) or metal cations (adverse organoleptic changes may occur) [3]; and use of lower temperatures, below 120 °C [13].

Some authors have demonstrated the effectiveness of hydrocolloid solutions in preventing the formation of acrylamide in various dried food products [14,15]. Hydrocolloids (such as proteins and polysaccharides) are hydrophilic polymers that have thickening, gelling, and emulsifying properties [16]. Champrasert et al. [17] studied the influence of polysaccharides, such as citrus pectin, sodium alginate, and chitosan, on the formation of acrylamide in chemical and food model systems subjected to heating using various methods (i.e., heating block and microwave). It has been shown that the most effective inhibitor of acrylamide formation, both in the chemical and food models, is alginate, followed by pectin. The least effective acrylamide inhibitor was chitosan. The authors [17] showed that the use of an appropriately low concentration of chitosan (0.1%) can increase the amount of acrylamide produced. A significant positive effect of the presence of pectin in a cookie recipe on the reduction of acrylamide was also demonstrated by Wang et al. [18]. The authors used the addition of various pectin preparations, indicating that the best effect in reducing the amount of AA was achieved by adding apple pomace preparation to the dough (AA reduction from 48% to 63%). An important observation by these authors [18] was, as demonstrated, that the presence of nonstarch polysaccharides (i.e., pectins), especially the preparation in the form of apple pomace, significantly reduced the bioavailability of AA in the gastrointestinal tract, which was, on average, 13% compared to the bioavailability of the control sample (>63%). In turn, Rossi Marquez et al. [19] showed that the application of a coating consisting of whey protein or pectin prepared in the presence of transglutaminase on the surface of potatoes was an effective way to reduce the acrylamide content of fried potatoes. Moreover, according to Suyatma et al. [15], the preblanching of banana slices and the pectin coating that was applied to the surface of the bananas caused a reduced amount of acrylamide to form during the frying of the banana chips. According to Zeng et al. [6], alginic acid and pectin are promising inhibitors of acrylamide formation in fried potatoes. One of the compounds that has gelling, thickening, and texture-forming properties is β-glucan [20]. β-Glucan is a polysaccharide consisting of D-glucose units linked by β-glycosidic bonds. The most common source of β-glucan are cereal grains, such as oats, wheat, and barley, as well as mushrooms of the basal and bagel classes [21]. Particular attention should be paid to the structure (β-(1,3/1,4)-D-glucan) and health properties of β-glucans derived from oats. The β-glucan content in oat grain is about 4–7% in whole grains and 6–9% in bran. Eighty percent of oat β-glucans are water soluble [22]. β-Glucans are characterized by anticancer activity, mainly against breast and colon cancer cells, and antioxidant and anti-inflammatory properties (due to the presence of phenolic compounds). In addition, β-glucans lower blood sugar levels, which can be used in the prevention and treatment of diabetes, and lower cholesterol levels and the associated risk of cardiovascular disease, prolonging cell viability. β-Glucans can also be used in the prevention and treatment of cardiovascular diseases. β-Glucans also have prebiotic properties; thus, they may also prevent colorectal and digestive system diseases [23]. Because of the gelling, thickening, and textural, as well as health-promoting, properties of β-glucans, there has been growing interest in the use of β-glucans in the food industry in recent years. β-Glucan preparations are used as thickening agents. In addition, they are also added to dairy and oatmeal drinks, yogurts, and ice cream, where they act as a bioactive agent, stabilizer, and texture enhancer [20].

In view of the above, this study was designed to explore the potential of oat fiber preparations in mitigating the formation of acrylamide during the production of dietary bread, specifically rusks The tests were carried out using optimal process conditions for obtaining this type of product, examining the effect of different contents of β-glucan fractions in the fiber preparations used (20% (OFP^20% β-glucans^) and 30% (OFP^30% β-glucans^)) and the effect of the concentration of these fiber preparations on their ability to reduce the amount of acrylamide formed in rusks. Simultaneously, the study aimed to shape their desired physicochemical properties, including water activity, color profile, and free carbohydrates and free asparagine compositions. By examining these parameters, we aimed to elucidate through which oat fiber are these aspects influenced and contribute to enhancing both product quality and consumer safety. Moreover, considering the few reports on the positive effect of hydrocolloids on reducing the amount of the carcinogen acrylamide that forms in food and the lack of studies involving β-glucans in this role, the direction of the research undertaken by the authors of the manuscript seems justified.

## 2. Results and Discussion

### 2.1. Effect of the Addition of the Different Concentrations of Oat Fiber Preparations on the Acrylamide Content

**Figure 1:**
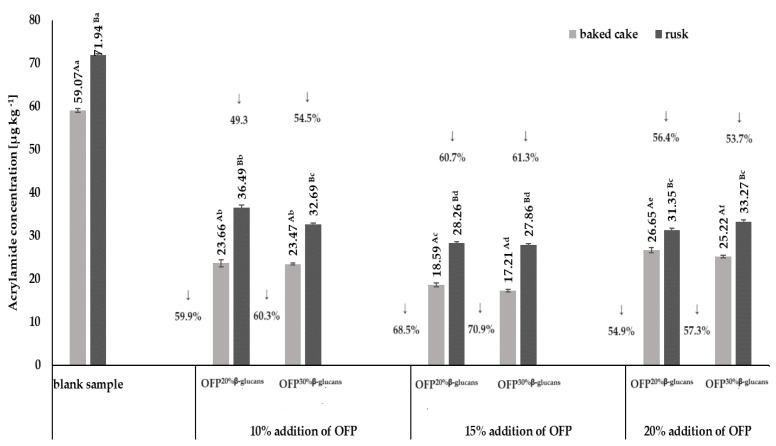
Changes in the acrylamide concentration in baked cakes and rusks depending on the type and concentration of the oat fiber preparation in the recipe composition. Blank sample-dough without the addition of an oat fiber preparation; OFP^20% β-glucans^-oat fiber preparation with a 20% β-glucans content; OFP^30% β-glucans^-oat fiber preparation with a 30% β-glucans content; uppercase letters-different letters within the same row indicate significant differences in the acrylamide concentration depending on the type of product (baked cake or rusk) for a given concentration of fiber preparation (*n* = 3; *p* ≤ 0.05); lowercase letters—different letters within the same row indicate significant differences in the acrylamide concentration depending on the concentration of the added fiber preparation for a given type of product (baked cake or rusk) (*n* = 3; *p* ≤ 0.05); data are presented as the mean ± SD.

The studies presented in this publication focused on the effects of different concentrations of an oat fiber preparation containing β-glucan at two different levels (20% (OFP^20% β-glucan^) and 30% (OFP^30% β-glucan^)) on acrylamide formation in yeast dough and rusks obtained from this dough. The results of the acrylamide content in baked goods and rusks are shown in Figure 1. 

The highest acrylamide content, both in baked goods and in rusks, amounting to 59.07 µg kg^−1^ and 71.94 µg kg^−1^, respectively, was found in the blank samples, i.e., without oat fiber. The acrylamide levels determined in the obtained blank samples of both baked goods and rusks did not exceed the reference levels for this compound, specified in the EU Commission Regulation of 20 November 2017, which are 300 µg kg^−1^ and 150 µg kg^−1^, respectively, for baked goods and rusks. It was shown that the presence of oat fiber in the recipe of the rusks resulted in a statistically significant reduction (*p* < 0.05) of acrylamide content, both in baked goods and in rusks, compared to its content in the control sample (i.e., without the addition of a fiber preparation). The greatest reduction in acrylamide content compared to the control sample was observed in both the baked goods (by ~70%) and rusks (by ~61%) obtained with the use of a 15% addition of OFP^20% β-glucans^ and OFP^30% β-glucans^ in the recipe. The acrylamide contents in the baked goods were at the levels of 18.59 µg kg^−1^ and 17.21 µg kg^−1^ and in the rusks at the levels of 28.26 µg kg^−1^ and 27.86 µg kg^−1^, obtained with the addition of OFP^20% β-glucans^ and OFP^30% β-glucans^, respectively. The acrylamide contents in the baked yeast doughs correlated with the amount of both OFP^20% β-glucans^ and OFP^30% β-glucans^ used in the recipe. The observed substantial negative correlation coefficients, quantified at R^2^ = −0.84 for baked cakes and R^2^ = −0.89 for rusks, substantiate this assertion. All three used oat fiber concentrations (10%, 15%, and 20%) showed a significant level of effectiveness in reducing the acrylamide content (*p* < 0.05), both in baked cakes and in rusks, on average by ~62% and ~56%, respectively. However, it was noticed that the addition of oat fiber at a concentration of 20% increased the acrylamide content in baked goods and in the rusks obtained from them, in relation to concentrations of oat fiber of 10% and 15%. However, it should be emphasized that the determined acrylamide contents in baked goods and rusks obtained with a 20% addition of oat fiber were significantly lower compared to its content in the samples without the addition of fiber. According to Al-Asmar et al. [24], the use of the addition of nonstarch polysaccharide preparations (i.e., pectin and chitosan) with water-binding properties for the production of potato French fries resulted in an approx. 40% reduction in the acrylamide content in these products. β-Glucans, which are the main component of the soluble oat fiber fraction in the used preparations, are also characterized by strong gelling and thickening properties, as well as the ability to bind water. The abovementioned properties of the β-glucans present in the oat fiber preparations used mean that, in their presence, starch is depolymerized to a much lesser extent into glucose, which is one of the precursors of acrylamide formation. This is reflected in the formation of less acrylamide in the baked goods and rusks. The final step in the production of the rusks was drying 1.5 cm thick slices of baked yeast dough at 150 °C for 22 min. On the basis of the obtained results (Figure 1), it was shown that the drying process also increases the acrylamide content in the final product, i.e., rusks. It was observed that, regardless of the concentration used and the type of oat fiber preparation, the process of drying slices of baked yeast dough increased the acrylamide content in the obtained rusks by an average of 40%. The data presented in Figure 1 show that the smallest increase (18%) in the acrylamide content in the process of drying slices of baked yeast dough was observed in the product with the addition of 20% OFP^20% β-glucans^, and the highest (62%) was in the case of bread with the addition of 15% OFP^30% β-glucans^. The reduction in acrylamide content observed with the use of an oat fiber preparation of OFP^20% β-glucans^ or OFP^30% β-glucans^ in the recipe for the rusks can be attributed to the interaction of functional groups present in the β-glucans chains with Maillard reaction precursors. The oat fiber preparations utilized, comprising varying proportions of β-glucan fractions, are distinguished by their molecular architecture, which features extensive chains replete with numerous chemical functional groups. This may increase the likelihood of interactions of the functional groups of β-glucan with amino acids or reducing sugars and, thus, reduce their availability in the nonenzymatic browning reaction and, therefore, reducing the formation of acrylamide during the baking of the dough and also in the drying process for the baked slices of yeast dough [25]. The obtained results on the acrylamide content in the baked yeast doughs and in the rusks suggest that oat fiber preparations with a 20% or 30% content of the soluble β-glucan fraction (OFP^20% β-glucans^ or OFP^30% β-glucans^) may be promising inhibitors of acrylamide formation in these types of food products. Also, Zeng et al. [6], who investigated the effect of eight preparations of water-soluble fiber on the amount of acrylamide formed in model starch systems, showed a reduction in acrylamide ranging from 30% to 50% in systems containing the addition of dietary fiber compared to the control sample. Similarly, Sansano et al. [14] demonstrated the protective effect of the addition of a soluble fraction of dietary fiber (SDF) on the amount of acrylamide formed in starch-based model systems that were heated at 170 °C. The results on the acrylamide content obtained by the authors showed reductions in this compound in the range from 59% to 85% in model systems obtained with the addition of a soluble fraction of dietary fiber in the amounts of 0.3% and 1.0%, respectively.

### 2.2. Effect of the Type and Concentration of the Oat Fiber Preparations on the Contents of Acrylamide Precursors

#### 2.2.1. Effect of the Type and Concentration of the Oat Fiber Preparations on the Contents of Free Sugars

Table 1 and Table 2 show the contents of free fructose, glucose, sucrose, and maltose and their sum in the raw yeast doughs before and after the fermentation process and in the baked yeast doughs and rusks, respectively.

The data presented in Table 1 show that among the carbohydrates determined in the raw yeast doughs, obtained with the addition of various concentrations of the oat fiber preparations, with 20% and 30% of their composition composed of a soluble fraction of β-glucans (OFP^20% β-glucans^ or OFP^30% β-glucans^), the largest share was fructose, the content of which was in the range of 21.09 mg g^−1^ d.m. in the dough with 15% OFP^30% β-glucans^ to 24.51 mg g^−1^ d.m. in the dough with 20% OFP^30% β-glucans^; then maltose, in the range of 23.23 mg g^−1^ d.m. in the dough without the addition of an oat fiber preparation (i.e., blank sample) up to 28.58 mg g^−1^ d.m. in the dough with 20% OFP^20% β-glucans^; and glucose in the range of 13.31 mg g^−1^ d.m. in the dough with 15% OFP^30% β-glucans^ to 17.93 mg g^−1^ d.m. in the dough with 20% OFP^30% β-glucans^. In the obtained raw yeast doughs, sucrose had the smallest share, which was in the range from 0.10 mg g^−1^ d.m. in the dough with 20% OFP^30% β-glucans^ to 0.76 mg g^−1^ d.m. in the dough with 15% OFP^20% β-glucans^.

One of the steps in the preparation of rusks is fermentation of the dough. The effect of this treatment is the leavening of the dough by yeast, which uses the simple free sugars and amino acids present in the dough, mainly asparagine. The result of this process is the formation of CO_2_. The analysis of the contents of free sugars in the raw yeast doughs, obtained with the addition of oat fiber preparations (OFP^20% β-glucans^ or OFP^30% β-glucans^) of various concentrations and without their addition, showed reductions in the contents of individual free sugars and in the sum of free sugars compared to their contents in the dough before fermentation. By analyzing the results presented in Table 1, it is observed that after the fermentation process, the contents of fructose, glucose, sucrose, and maltose were 4%, 10%, 27%, and 23% lower, respectively, compared to their contents in the doughs before the fermentation process. 

The analysis of the carbohydrates profile of the baked yeast cakes, depending on the concentration of the type of oat fiber preparation used (OFP^20% β-glucans^ and OFP^30% β-glucans^), showed that the baked goods obtained with 15% OFP^20% β-glucans^ had the highest contents of fructose and sucrose. In turn, the highest contents of glucose and maltose were determined in the baked goods obtained with a 10% addition of this preparation (Table 2). The lowest contents of fructose, glucose, and maltose were found in the yeast dough obtained without the addition of an oat fiber preparation (i.e., control sample). Moreover, no sucrose was detected in this baking (Table 2). 

A comparative analysis of the sugar content of the raw yeast doughs and in the baked goods showed a decrease in the content of each type of determined carbohydrate in all baked yeast cakes. In the analyzed baked goods, fructose was reduced in the range from 0.4% to 46.0%, respectively, in the bread obtained with a 15% share in the recipe composition of the OFP^30% β-glucans^ preparation and in the yeast dough without oat fiber (i.e., control sample). The glucose content in the baked goods decreased in relation to its content in the raw doughs, ranging from 2.0% to 62.0%, respectively, in the bread obtained with a 10% share in the recipe composition of the OFP^20% β-glucans^ preparation and in the bread obtained with a 15% share in the recipe composition of OFP^30% β-glucans^.

In the obtained baked yeast doughs, the sucrose content was reduced by 43.0% on average, compared to its content in raw doughs. The highest reduction in sucrose content was observed in baked goods obtained with 20% addition of OFP^30% β-glucans^ and in baked goods without the addition of oat fiber preparations (100.0%), and the lowest in baked goods obtained with 15% addition of OFP^20% β-glucans^ (8.0%) (Table 2).

During the baking process, degradation of maltose also occurred. Its content in the baked goods was, on average, 71.0% lower than in the raw doughs. The highest reduction in the content of maltose was observed in the yeast cakes obtained without the addition of an oat fiber preparation (i.e., control sample) (93.0%), the lowest in the postbaked cakes obtained with 10% and 20% additions of OFP^20% β-glucans^ (60.0%).

As reported in the literature [26], reducing sugars, mainly fructose and glucose, are one of the substrates necessary for the formation of acrylamide in thermally processed food products. The authors of the publication determined the correlation between the content of the individual sugars present in the obtained raw yeast dough and the content of acrylamide in the baked goods and rusks obtained with different concentrations of oat fiber preparations OFP^20% β-glucans^ and OFP^30% β-glucans^, differing in fiber content. The free sugars present in the raw yeast doughs and in the baked cakes correlated differently with the amount of acrylamide formed in the baked goods and rusks, respectively. Significant negative correlation coefficients were observed between the concentrations of glucose, maltose, and the total sugars in the raw yeast doughs and baked products and the acrylamide content in the baked yeast cakes and rusks. These correlations were evident in the products formulated with varying concentrations of OFP^20% β-glucans^ and OFP^30% β-glucans^, yielding R^2^ values of −0.70, −0.90, and −0.78 for the yeast cakes and −0.86, −0.95, and −0.96 for the rusks, respectively. Regardless of the type of oat fiber preparation used, along with an increase in its concentration in the recipe, an increase in the contents of glucose and maltose and the sum of sugars in the raw yeast doughs and baked goods was observed, with a simultaneous reduction in the amount of acrylamide formed in the baked goods, as well as in the resulting rusks. The correlation between the fructose content in the raw yeast doughs and the amount of acrylamide formed during the baking process varied depending on the type of oat fiber preparation used in the recipe composition of the yeast doughs. A marginal negative correlation was observed between the fructose concentration in the raw yeast dough and the acrylamide formation in the baked yeast cake prepared with OFP^20%^ β^-glucans^ (R^2^ = −0.23), while a slight positive correlation was noted between the fructose content and acrylamide production in the yeast cake baked with OFP^30% β-glucans^ (R^2^ = 0.46). On the basis of the calculated correlation coefficients, it was observed that in the case of the baked goods obtained with OFP^20% β-glucans^, along with the increase in its concentration in the recipe, an increase in the fructose content in the raw dough was observed with a simultaneous decrease in the amount of acrylamide formed. Conversely, for the baked products formulated with OFP^30% β-glucans^, an inverse relationship was noted, wherein an elevation in its concentration in the recipe corresponded to a concurrent reduction in both the fructose content in the raw dough and the acrylamide level in the finished baked good. On the other hand, in the case of rusks, the amount of fructose present in the baked goods was strongly negatively correlated with the amount of acrylamide formed during the process of drying slices of baked yeast dough, as evidenced by the correlation coefficients of R^2^ = −0.89 and R^2^ = −0.97 determined for the rusks obtained with the addition of OFP^20% β-glucans^ and OFP^30% β-glucans^, respectively.

#### 2.2.2. Effect of the Type and Concentration of the Oat Fiber Preparations on the Content of Free Asparagine

**Figure 2:**
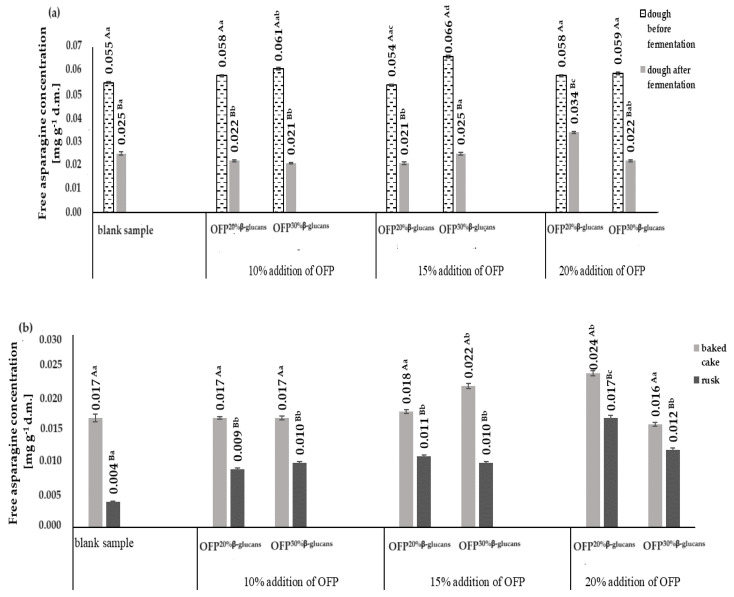
Free asparagine concentrations in the doughs before and after the fermentation process (**a**) and in the baked cakes and rusks (**b**) depending on the type and concentration of the oat fiber preparation used in the recipe composition. Blank sample-dough without the addition of an oat fiber preparation; OFP^20% β-glucans^-oat fiber preparation with a 20% β-glucans content; OFP^30% β-glucans^-oat fiber preparation with a 30% β-glucans content; uppercase letters-different letters within the same row indicate significant differences in the acrylamide concentration depending on the type of product (baked cake or rusk) for a given concentration of fiber preparation (*n* = 3; *p* ≤ 0.05); lowercase letters-different letters within the same row indicate significant differences in the acrylamide concentration depending on the concentration of the added fiber preparation for a given type of product (baked cake or rusk) (*n* = 3; *p* ≤ 0.05); data are presented as the mean ± SD.

Figure 2a,b show the content of free asparagine in raw yeast dough, before and after its fermentation, and in baked yeast dough and rusks, respectively. 

The contents of free asparagine in the raw yeast doughs ranged from 0.054 mg g^−1^ d.m. up to 0.066 mg g^−1^ d.m. The highest content of free asparagine was found in the raw yeast dough obtained with 15% OFP^30% β-glucans^, while the lowest content was in the dough obtained with 15% OFP^20% β-glucans^. The content of free asparagine decreased as a result of the fermentation of the yeast dough (on average by 58.0%). The greatest reduction (65.0%) in the free asparagine content during the fermentation process was observed in the dough with 10% OFP^30% β-glucans^, while the lowest (41.0%) was in the dough with 20% OFP^20% β-glucans^ (Figure 2a). These results are correspondent with the findings of Fredriksson et al. [26], who in their studies monitored the content of free asparagine in bread dough subjected to the fermentation process with yeast. The authors observed a 40.0% reduction in the content of free asparagine in the dough subjected to a 1 h fermentation process. In the dough fermentation process, free asparagine is the main source of nitrogen necessary for yeast growth.

The baked yeast cakes were characterized by a content of free asparagine in the range of 0.016 mg g^−1^ d.m. up to 0.024 mg g^−1^ d.m. (Figure 2b). The lowest content of free asparagine was found in baked goods obtained with a 20% addition of OFP^30% β-glucans^. The highest content of this amino acid was found in the baked goods obtained with a 20% addition of OFP^20% β-glucans^. During the baking process, the content of free asparagine in the cakes was reduced in the range from 12.0% to 32.0%, respectively, in the cakes obtained with 15% OFP^30% β-glucans^ and in the cakes without an oat fiber preparation (i.e., control sample). The reduction in the content of free asparagine in yeast dough is due to the fact that, in addition to reducing sugars, it is a substrate necessary for the formation of the carcinogenic compound acrylamide [27]. The degree of the reduction in the free asparagine present in raw yeast cakes during the baking process was strongly positively correlated with the amount of acrylamide formed, as evidenced by the correlation coefficient of R^2^ = 0.79. This indicates a lower use of asparagine in the acrylamide formation process and may be one of the reasons for the formation of lower amounts of acrylamide (AA) in the baked goods and biscuits obtained with the addition of the oat fiber preparations OFP^20% β-glucans^ and OFP^30% β-glucans^. A correlation was established between the level of free asparagine quantified in both the raw and baked yeast cakes and the acrylamide content in the corresponding baked goods and rusks, which were prepared using the oat fiber formulations OFP^20% β-glucans^ and OFP^30% β-glucans^. The free asparagine present in the raw yeast doughs and baked doughs correlated differently with the amount of acrylamide formed in the baked goods and rusks. A modest positive correlation was discerned between the concentration of free asparagine in the raw yeast doughs, formulated with varying ratios of OFP^20% β-glucans^ and OFP^30% β-glucans^, and the resultant acrylamide production following their baking. This correlation is evidenced by the calculated coefficients, registering at R^2^ = 0.10 and R^2^ = 0.42, respectively. On the basis of the calculated correlation coefficients, it can be seen that with the increase in the share of oat fiber in the recipe for the yeast doughs, a decrease in the content of free asparagine in the raw doughs was observed, with a simultaneous reduction in the amount of acrylamide formed during the baking process.

Furthermore, a slight negative correlation was observed between the level of free asparagine in the baked yeast doughs, prepared with varying concentrations of OFP^20% β-glucans^ and OFP^30% β-glucans^, and the acrylamide content produced during the drying process for the bread slices. This relationship is substantiated by the correlation coefficients of R^2^ = −0.43 and R^2^ = −0.36, respectively. With the increase in the share of oat fiber in the recipe for the yeast doughs, an increase in the content of free asparagine in the baked cakes was observed, while the amount of acrylamide produced during the process of drying the bread slices decreased.

Correlations between the amounts of free reducing sugars (i.e., fructose, glucose, and maltose) and the free asparagine in the raw yeast doughs obtained with different concentrations of the oat fiber preparations were also determined. In the case of the yeast dough obtained with the addition of an oat fiber preparation with a 20% content of the β-glucan fraction (OFP^20% β-glucans^), correlation coefficients were determined, respectively, at R^2^_(fructose/asparagine)_ = 0.85, R^2^_(glucose/asparagine)_ = 0.69, and R^2^_(maltose/asparagine)_ = 0.49. In turn, for the yeast dough containing in its recipe composition different concentrations of oat fiber with a 30% content of the β-glucan fraction (OFP^30% β-glucans^), the determined correlation coefficients were as follows: R^2^_(fructose/asparagine)_ = 0.99, R^2^ _(glucose/asparagine)_ = 0.93, and R^2^_(maltose/asparagine)_ = 0.91. On the basis of the calculated correlation coefficients, it was observed that with the increase in the share of the oat fiber in the recipe for the yeast doughs, the contents of free asparagine and reducing sugars (fructose, glucose, and maltose) in the raw doughs decreased simultaneously, which resulted in the formation during the baking process of smaller amounts of acrylamide. In addition, it is worth emphasizing that stronger correlations between free reducing sugars and free asparagine, as evidenced by high correlation coefficients, were found in the raw yeast dough obtained with the addition of oat fiber with a higher concentration of the β-glucan fraction (OFP^30% β-glucans^). This is reflected in the formation of lower amounts of acrylamide during the baking process for the cakes with the addition of oat fiber with a higher content of the β-glucan fraction (OFP^30% β-glucans^).

### 2.3. Influence of the Type and Concentration of the Oat Fiber Preparations on the Color of the Yeast Cakes and Rusks

Color is an important quality parameter of most food products, determining their acceptance and influencing consumer preferences. Measurement results of color parameters such as L*, a*, and b* and their derivatives, i.e., total color difference from the control sample (∆E) and browning index (BI) for samples of yeast cakes and rusks, obtained with various additions of oat fiber preparations, differing in the content of water-soluble β-glucan fractions (OFP^20% β-glucans^ and OFP^30% β-glucans^), are presented in Table 3.

The brightness analysis (L*) showed that most of the yeast cakes and rusks obtained with the use of oat fiber preparations in the recipe composition were lighter in comparison with the yeast cakes and rusks obtained without the addition of these preparations, which served as the reference sample in the study (Table 3). In almost all cases, the ΔE parameter was higher than three; therefore, the color change in the yeast cakes and rusks obtained with the addition of oat fiber preparations, compared to those obtained without fiber, was visible and obvious to the human eye. Exceptions were made for baking with 10% OFP^20% β-glucans^ and 20% OFP^30% β-glucans^, for which the ΔE was only 1.20 and 1.55, respectively. This means that the products were only slightly lighter compared to the baked yeast cakes and rusks obtained without the addition of oat fiber preparations. The highest values of ΔE, at the level of 7.97 and 9.78, were shown for the baked yeast cake with 15% OFP^30% β-glucans^ and for rusk with 15% OFP^20% β-glucans^, respectively (Table 3). This indicates that these samples were the brightest of all analyzed products. The analysis of the results presented in Table 3 in terms of the intensity of the red and yellow in the obtained yeast cakes and rusks showed that the baked goods obtained with a 15% addition of OFP^20% β-glucans^ and OFP^30% β-glucans^ had the lowest concentrations of yellow and red pigments among the tested samples of baked goods and rusks. It was shown that the use of the oat fiber preparations OFP^20% β-glucans^ and OFP^30% β-glucans^ in the recipe composition of the yeast doughs, regardless of their concentration, reduced the intensity of the yellow and red pigments, both in the yeast cakes and rusks. On the basis of the color parameters determined for the obtained yeast cakes and rusks, the browning index (BI) was calculated for each sample. The browning index of the obtained yeast cakes and rusks ranged from 25.82 to 39.47 and from 28.47 to 46.88 (Table 3), respectively. The highest browning index (BI) was found in both the baked goods and rusks that did not contain oat fiber preparations in their recipe composition. The lowest browning Index (BI) was found for the baked goods obtained with a 15% addition of OFP^30% β-glucans^ and rusks with a 15% addition of OFP^20% β-glucans^ (Table 3). It was shown that the use of oat fiber preparations in the recipe composition of the yeast doughs, regardless of the content of the water-soluble β-glucan fraction in them, decreased in the browning index for the obtained baked goods and rusks compared to the control samples, i.e., without the addition of fiber preparations. 

As reported in the literature, acrylamide is one of the end products of the Maillard reaction, i.e., a reaction that produces the desired flavor and browned color of the heat-treated products [28]. In view of the above, the authors of the publication determined the correlations between the color parameters L*, a*, b*, and BI, and the acrylamide content in the yeast cakes and rusks obtained with the addition of an oat fiber preparation, differing in the content of the water-soluble β-glucan fraction. During the analysis of the values of the correlation coefficients between the color parameters and the acrylamide content in the yeast cakes and rusks obtained with the addition of various concentrations of the oat fiber preparation, a differentiation was observed depending on a given color parameter. Substantial positive correlation coefficients were identified between the intensity of the yellow coloration (b*) and the acrylamide content in the yeast cakes and rusks prepared with varying concentrations of OFP^20% β-glucans^ and OFP^30% β-glucans^. These coefficients were quantified at R^2^ = 0.72 and R^2^ = 0.84 for the yeast cakes and R^2^ = 0.92 for both concentrations in the rusks. Regardless of the type of oat fiber preparation used, along with an increase in its concentration in the recipe, a decrease in the intensity of the yellow color was observed, with a simultaneous decrease in the acrylamide concentration both in the baked goods and in the rusks obtained from them. The computed correlation coefficients between the browning index (BI) and the acrylamide content in the yeast cakes and rusks revealed a pronounced positive correlation for these parameters. These correlations were quantified as R^2^ = 0.73 and R^2^ = 0.93 for the yeast cakes and R^2^ = 0.91 and R^2^ = 0.89 for the rusks, corresponding to the addition of OFP^20% β-glucans^ and OFP^30% β-glucans^, respectively. In this case, a decrease in the browning index (BI) was observed with the simultaneous reduction in acrylamide (AA) formed with the increase in the concentration of the oat fiber preparations in the yeast dough recipe.

Pedreschi et al. [29] showed a strong linear correlation between the acrylamide concentration and the values of lightness (L*) and red color intensity (a*) in potato chips, as evidenced by their calculated correlation coefficients, R^2^ = 0.79 and R^2^ = 0.83, respectively. In our case, no strong linear correlation was found between the L* and a* parameters and the acrylamide content in the obtained baked goods and rusks, along with an increase in the concentration of the oat fiber preparations in the recipe, regardless of their type (OFP^20% β-glucans^ and OFP^30% β-glucans^). In the case of the relationship between the intensity of red pigments (a*) and the amount of acrylamide (AA) formed, it was observed that with the increase in the concentration of the oat fiber preparations in the recipe, a simultaneous decrease in the concentration of red pigments and acrylamide took place, as evidenced by the positive correlation coefficients of R^2^ = 0.53 and R^2^ = 0.54 for the yeast cakes and rusks, respectively, regardless of the type of oat fiber preparation used (OFP^20% β-glucans^ and OFP^30% β-glucans^). Analyzing the relationship between the color parameter (L*) and the concentration of acrylamide in the obtained yeast cakes and rusks, negative values of the correlation coefficients were determined, on average, at the level of R^2^ = −0.61 and R^2^ = −0.25, respectively for OFP^20% β-glucans^ and OFP^30% β-glucans^. In the case of this relationship, with the increase in the concentration of the oat fiber preparations in the recipe, an increase in the value of the L* color parameter was observed, reflecting the brightening of the color of the obtained products with a simultaneous decrease in the amount of acrylamide (AA) formed. Summarizing the determined correlation coefficients between the color parameters and the amount of acrylamide formed in both the yeast dough baked goods and in the rusks obtained from them, a strong correlation between the intensity of the yellow pigments and the browning index (BI) and the amount of acrylamide (AA) formed during the baking process for the yeast dough and then the drying of the slices of the yeast cakes is indicated. In view of the above, it can be concluded that the browning index (BI) can be a very effective indicator of the amount of acrylamide (AA) formed in the baking process and can serve as a tool to eliminate very dark or burnt products in order to avoid exposure to or consumption of large amounts of acrylamide (AA) with food.

### 2.4. Water Activity

**Figure 3:**
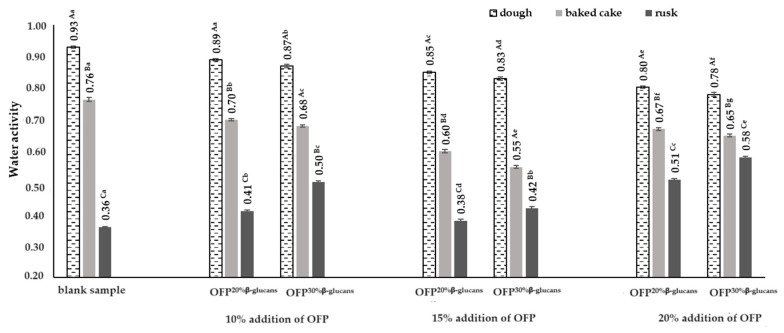
Water activity in the doughs, baked cakes, and rusks depending on the type and concentration of the oat fiber preparation used in the recipe composition. Blank sample-dough without the addition of an oat fiber preparation; OFP^20% β-glucans^-oat fiber preparation with a 20% β-glucans content; OFP^30% β-glucans^-oat fiber preparation with a 30% β-glucans content; uppercase letters-different letters within the same row indicate significant differences in the water activity depending on the type of product (dough, baked cake, or rusk) for a given concentration of the fiber preparation (n = 3; *p* ≤ 0.05); lowercase letters-different letters within the same row indicate significant differences in the water activity depending on the concentration of the added fiber preparation for a given type of product (dough, baked cake, or rusk) (n = 3; *p* ≤ 0.05); data are presented as the mean ± SD.

The water activity (a_w_) determines the active part of the water content of a product (i.e., “free water”), as opposed to the total water content, which also contains “bound water”. The value of this parameter has a decisive impact on a number of processes taking place in food products, such as change in color, taste, aroma, production of food poisons, spoilage (i.e., expiry date), or loss of nutrients. Water activity is also one of the factors determining the speed of the Maillard reaction in food products [30]. In the obtained raw yeast doughs, the water activity was determined in the range from 0.78 to 0.93 (Figure 3).

The dough that did not contain an oat fiber preparation in its recipe composition was characterized by the highest activity of water. The lowest water activity was determined in the dough obtained with the addition of 20% OFP^30% β-glucans^ in the recipe. The use of an oat fiber preparation in the recipe composition of the yeast doughs, regardless of its concentration and the content of the water-soluble β-glucan fraction in them, resulted in a significant reduction in water activity in the raw doughs compared to the control sample, i.e., without the addition of an oat fiber preparation (Figure 3). Similar results were obtained by Havrlentová et al. [31], who showed that the addition of oat B-glucans to the bread dough recipe reduced the water activity value by 0.046 in the dough with the addition of this hydrocolloid compared to the water activity in the control dough. Moreover, Havrlentová et al. [31] showed that the addition of B-glucans isolated from barley, wheat, and rye to bread dough resulted in an increase in the water activity of the dough with the addition of these hydrocolloids by 0.002–0.012 compared to the water activity of the control dough. According to Vasanthan and Temelli [32], differences in the water activity of the doughs with the addition of B-glucans from different cereals may be caused by the different molecular weights and structures of cereal β-D-glucans. Also, Fernandes and de las Mercedes Salas-Mellado [33] and Nourmohammadi and Peighambardoust [34] showed that the use of chia seed mucilage in low-fat cakes reduces the free water content and, consequently, reduces the water activity, increasing storage durability of cakes). Hydrocolloids have the ability to absorb water and, at the same time, are able to limit its migration from the product during its thermal treatment. Among other things, research conducted by Bartkiene et al. [35] showed that the use of psyllium husk gel in the recipe composition of bread reduces the process of moisture migration in bread. At the same time, it correlated with the lower acrylamide content in the bread prepared with psyllium husk gel. The authors achieved a significant reduction in the amount of acrylamide in bread with the addition of psyllium husk gel due to its ability to limit the migration of water from the product during baking. The obtained research results indicate the possibility of using hydrocolloids to obtain safer finished products, i.e., bakery products.

The baking process reduced the water activity in the yeast cakes, regardless of the type of oat fiber preparation used. The water activity in the baked yeast doughs ranged from 0.55 to 0.76. The greatest reductions in the water activity, by 34% and 29%, in relation to the raw doughs were recorded in the doughs with a 15% addition of OFP^30% β-glucans^ and OFP^20% β-glucans^, respectively. The water activity in the rusks ranged from 0.36 in the product without the addition of an oat fiber preparation to 0.51 in the product with 20% OFP^20% β-glucans^ and was 53% and 11% lower, respectively, compared to the water activity in the baked cakes. According to Zokaei et al. [36], water activity ranging from 0.6 to 0.8 is optimal for the Maillard reaction. Lowering the water activity below 0.5 reduces the Maillard reaction rate, which is caused by the reduced mobility of the molecules and an increase in the matrix viscosity. Moreover, increasing the water activity above 0.8 also causes a decrease in the Maillard reaction rate, as a result of the dilution of the necessary substrates for its occurrence. According to Mustafa et al. [37] and Bassama et al. [38], water activity (a_w_) is one of the factors influencing the amount of acrylamide formed during the thermal processing of food products. According to the mentioned authors, the water activity in the model systems and food products is inversely correlated with the amount of acrylamide formed, which means that with the increase in the water activity in the tested food products, the amount of formed acrylamide decreases. For the results on the water activity and acrylamide content in the baked goods and rusks obtained in the study, correlation coefficients were calculated to determine the relationship between these parameters. On the basis of the calculated correlation coefficients, strong and directly proportional relationships were found between the water activity (a_w_) and the acrylamide content, both in the baked goods and rusks. It was found that with an increase in the concentration of oat fiber preparations in the recipe of the yeast doughs, the water activity (a_w_) decreased, with a simultaneous decrease in the amount of acrylamide formed, both in the baked goods and the rusks obtained from them, as evidenced by the correlation coefficients of R^2^ = 0.73 and R^2^ = 0.84, respectively. Additionally, it was observed that substituting an oat fiber preparation with a 30% water-soluble β-glucan fraction (OFP^30% β-glucans^) for one with a 20% β-glucan fraction (OFP^20% β-glucans^) in the yeast dough recipes resulted in a more pronounced correlation between the water activity (a_w_) and acrylamide (AA) content in the baked products. This is demonstrated by a correlation coefficient of R^2^ = 0.77, which is 13% higher than the coefficient (R^2^ = 0.68) determined for the baked goods produced with varying concentrations of the oat fiber preparation containing 20% β-glucans (OFP^20% β-glucans^).

## 3. Materials and Methods

### 3.1. Chemicals

The reference standard of asparagine was purchased from Sigma-Aldrich. The standards of saccharides (glucose, fructose, maltose, ribose, and sucrose) were supplied by Fluka. Acrylamide (99%) was procured from Merck, and 2,3,3-d3-acrylamide (>98%) was obtained from Cambridge Isotope Laboratories, Inc. (Andover, MA, USA). Acetonitrile (99.9%) was purchased from Sigma-Aldrich. All other reagents were of analytical grade and supplied by POCH (Gliwice, Poland).

### 3.2. Preparation of Rusks

The following ingredients were used to obtain rusks: wheat flour, crystal sugar, compressed yeast, fat (margarine “Kasia”), whole milk, water, salt—all from a local market—oat fiber preparation with a 20% β-glucan content (OFP^20% β-glucans^), and oat fiber preparation with a 30% β-glucan content (OFP^30% β-glucans^) (both were purchased from Brenntag Polska Sp. z o.o.), all in the proportions stated below. Rusk dough formulation (g 100 g^−1^): wheat flour (0.5% ash)—48.70; fat—4.68; yeast—1.87; crystal sugar—7.49; milk—28.09; water—8.80; and salt—0.37 

The preparations OFP^20% β-glucans^ and OFP^30% β-glucans^ were added to dough in the amounts of 10, 15, and 20 g 100 g^−1^ based on the weight of all of the components, substituting flour. 

The yeast dough was prepared with the use of a three-phase method. To prepare leaven, 50% flour, 80% milk, and all yeast from a recipe were used. The mixture was blended in a lab-scale dough blender (Bosch ProfiMixx 46, Slovenia). The leaven was fermented at 31 °C with a relative air humidity of 45% for 30 min. To a risen leaven, a sugar–water solution was added, as well as the rest of the milk, flour, and salt. After 5 min of blending, heated fat was added, as well as the preparation OFP^20% β-glucans^ or OFP^30% β-glucans^. Afterward, the dough was mixed for 5 more minutes. Ready dough was fermented once again as previously described. The risen yeast dough was transferred to a greased sheet and subjected to final fermentation in a fermentation chamber at 31 °C with a relative air humidity of 45% for 15 min. Fermented dough was baked in an electric oven (Ariston C 3 VP6) at 180 °C for 40 min. After baking, the dough was cooled down and laid out for 24 h at 20 °C. The dough was cut into 1.5 cm thick slices, placed on grids, and dried in a dryer at 150 °C for 22 min. 

#### 3.2.1. Analyses Made for the Dough

##### Water Activity

The measurement of the water activity (a_w_) in a dough was performed with the use of a Hygropalm AW-1 (Rotronic AG, Bassersdorf, Switzerland). The dough and crumbled cake and rusk (circa 2 g) were placed in a WP-40 vial and left in the closed vial for around 30 min at 23 °C. Then, the opened vial was put on the plate of the device and closed with an AW-DIO probe.

##### Carbohydrate Content 

The contents of carbohydrates in a dough were determined using high-performance liquid chromatography (HPLC) by mean of Shodex NH2P-50 series columns [39], with our own modifications. 

A final ground sample (2 g with an accuracy of 0.0001 g) was weighed into a 50 mL centrifuge tube. Then, 15 mL of hot ultrapure water at 58 °C was added to extract sugars by shaking for 45 min. After centrifuging at 3024× *g* for 20 min and 20 °C, the supernatant was decanted and defatted twice with 10 mL of hexane. The defatted sample was then deproteinized by adding to it 0.1 mL of Carrez I and II reagents and centrifuging (3024× *g*, 10 min, 20 °C). After centrifugation, the supernatant was quickly decanted and filtered through a nylon syringe filter with a diameter of 0.22 μm to the autosampler vials and subjected to a chromatographic analysis.

The filtrate was analyzed for the free sugar contents using a UHPLC + Dionex UltiMate 3000 system (Thermo Fisher Scientific Inc., Waltham, MA, USA). Sugars were separated on a Shodex Asahipak NH2-50 4E (4.6 × 250 mm, 5 μm) (Shodex, Japan) at 30 °C and under isocratic conditions using an acetonitrile/water (70/30 *v*/*v*) solution as an eluate at a flow rate of 1.0 mL min^–1^. The peaks were detected by a refractive index detector Shodex-RI-101 (Shimadzu, Japan). Glucose, sucrose, maltose, and fructose were identified by comparing their retention times with reference standards. Quantification was carried out using an external standard method.

##### Free Asparagine Content Using an Amino Acid Analyzer

The content of free asparagine in a dough was determined according to Commission Directive 98/64/EC [40].

A total of 1 g of the ground sample was weighed into a 50 mL centrifuge tube with an accuracy of 0.0001 g, 10 mL of an extraction mixture composed of 0.1 mol L^−1^ HCl containing 2% thiodiglycol (2,2’-thiodiethanol, TDE) was added, and the tube was inserted into a shaking water bath and shaken for 60 min at 20 °C. After the completion of the reaction, the samples were centrifuged (3024× *g*, 10 min, 20 °C) and then defatted twice with hexane. A total of 2 mL of the defatted solution obtained above were collected into a glass vial. With continuous stirring using a magnetic stirrer, 1 mL of sulfosalicylic acid was added, and the whole was stirred for another 5 min. After this time, the solution was transferred to plastic tubes and centrifuged (3024× *g*, 5 min, 20 °C). The supernatant was collected and treated with 2 mL of buffer at pH 2.2. Then, the sample was filtered through a nylon membrane filter with 0.22 μm pore size into the autosampler vials and subjected to a chromatographic analysis.

The separation and quantitative analysis of free asparagine were carried out using a BIOCHROM 30+ amino acids analyzer. The identification of the separated asparagine was conducted based on the comparison of the retention times of the chromatographic fraction coming from the samples and solution of the reference standard. The quantitative determination of free asparagine present in the analyzed material was carried out by comparing the peak area of the tested amino acid in a sample with the surface area of the corresponding reference standard.

#### 3.2.2. Analyses Made for Baked Cakes and Rusks

##### Water Activity

The measurement of water activity (a_w_) in the baked cakes and rusks was performed in accordance with the methodology described in Section 3.2.1. 

##### Carbohydrate Content

The determination of the contents of carbohydrates in the baked cakes and rusks was performed in accordance with the methodology described in Section 3.2.1.

##### Free Asparagine Content Using an Amino Acid Analyzer

The determination of the content of free asparagine in the baked cakes and rusks was performed in accordance with the methodology described in Section 3.2.1.

##### Color Measurement in the CIE L*a*b* System

The measurement of the color of the baked cakes and rusks was performed using an automatic Konica Minolta colorimeter CR-400 with Spectra Magic NX 1.3 software (Osaka, Japan). Prior to the measurements, the equipment was calibrated using a standard white tile. The color of ground baked cake and rusk samples was measured in triplicate at several points for each sample. The color parameters (i.e., L*—brightness (from 0—black to 100—white), a*—(from (−50)—green to 50—red), b*—(from (−50)—blue to 50—yellow), and dE value, equal to the square root of [(dL*)2 + (da*)2 + (db*)2], characterizing the total change in color were measured. The obtained values were equivalent to the total color difference, whether obvious or not to the human eye, according to Bodart et al. [41]: dE* < 1, color differences are not obvious to the human eye; 1 < dE* < 3, minor color differences could be appreciated by the human eye depending on the hue; and dE* > 3 color differences are obvious to the human eye.

The browning index (BI) of the baked cakes and rusks was determined according to Wen and Chih [42].

##### Acrylamide Content

Acrylamide was quantified in the baked cakes and rusks with GC-MS/MS after derivatization using GC-MS/MS analysis parameters according to Mojska [43] and Soares and Fernandes [44].

The extraction and analysis of acrylamide were performed in accordance with the procedure described in a previous study by Miśkiewicz et al. [45].

The limit of detection (LOD) and limit of quantification (LOQ) for the method were calculated using the calibration curve parameters. In this case, the detection limit was 2.5 μg kg^−1^, and the limit of quantification was set at 5 μg kg^−1^. The recoveries were determined by adding 50 µg L^−1^ of the acrylamide standard solution to the sample. Average recoveries ranging from 73 to 89% were obtained.

### 3.3. Statistical Analysis

Each batch of rusks was baked three times. All analyses were carried out in triplicate; an average value and standard deviation were given as a result. The significance of differences was determined using t-Tukey’s test. The results at a significance level *p* < 0.05 were accepted as statistically significant. The results of the statistical evaluation are shown in the tables and figures, and the results differing statistically are labeled with different letters.

## 4. Conclusions

The obtained research results fill the gap regarding the continuous search for ways to reduce the dangerous carcinogen acrylamide in selected groups of food products. The tested product (i.e., rusks) is a relatively specific product both in terms of the method of its preparation and its intended use for selected groups of consumers (people on diets and convalescents). The conducted research is the first attempt to answer whether there is the possibility of reducing acrylamide in rusks. Additionally, for the first time, a very positive effect of polysaccharides, such as oat β-glucans, on the reduction of acrylamide in rusks was demonstrated.

On the basis of the obtained results, it was shown, for the first time, that the use of soluble oat fiber preparations with different contents of β-glucan fraction (20% and 30%) in the recipe composition of rusks allows for a statistically significant reduction in the content of acrylamide formed in the process of achieving these products. The greatest reductions in the acrylamide content (i.e., by 70% in baked cakes and by 61% in rusks) were possible to achieve with the use of a 15% addition of the oat fiber preparation, regardless of the share of the β-glucan fraction in its composition.

It should be emphasized that the use in the recipe composition of rusks, oat fiber preparations with different contents of β-glucan fraction allowed for a simultaneous significant reduction in acrylamide in the product and, additionally, the product was enriched with a soluble fiber fraction with many documented health properties.

It also seems important that along with the increase in the share of fiber preparations in the yeast dough recipe, a decrease in the content of free asparagine and reducing sugars (fructose, glucose, and maltose) in the raw dough was observed, which resulted in the formation of smaller amounts of acrylamide during the baking process. In addition, it is worth noting that stronger correlations between free reducing sugars and free asparagine were demonstrated in raw yeast dough obtained with the addition of an oat fiber preparation containing a higher concentration of β-glucan fraction, i.e., 30%. This association was manifested in reduced acrylamide formation during the baking of cakes incorporating oat fiber preparations containing a 30% fraction of β-glucans.

The authors of the manuscript showed a significant effect of the oat fiber preparations on shaping the color of the finished products. The use of oat fiber preparations in the amount of 15% in the yeast cake recipe, regardless of the concentration of the β-glucan fraction in them, reduced the intensity of yellow and red pigments, both in yeast cakes and in rusks. In addition, fiber preparations at this concentration reduced the browning index (BI) in the obtained baked goods and rusks to the greatest extent, compared to the control samples, i.e., without the addition of fiber preparations.

It was shown that there is a strong correlation between the intensity of the yellow pigments and the browning index (BI) and the amount of acrylamide (AA) formed during the baking process of the yeast dough and then drying the slices of yeast cakes. On this basis, it can be concluded that the browning index (BI) can be a very effective indicator of the amount of acrylamide (AA) in the finished product, i.e., in rusks.

Water activity is also one of the factors determining the speed of the Maillard reaction in food, one of the products of which is acrylamide. The authors of the manuscript also showed that oat β-glucans significantly affect the water activity parameter. In turn, this parameter has been shown to be strongly correlated with the content of acrylamide, both in the baked goods and rusks. Along with the decrease in the water activity value, a decrease in the acrylamide content in the finished product was observed. This is determined by the ability of hydrocolloids to water absorption, which limits the migration of water from the product during the baking process. The best effect in this respect was achieved when 15% addition of both types of tested oat fiber preparations were used in the rusk’s recipe.

The use of oat fiber preparations differing in the content of the β-glucan fraction as a factor reducing the acrylamide content by up to 70%, among others, in a dietary product such as rusks, turns out to be a more desirable solution compared to the use of, for example, chickpea protein [45]. In this case, using model systems for research, it was possible to reduce acrylamide by an average of 50%.

It should be emphasized that at the same time as showing the significant role of oat fiber preparations with different contents of β-glucan fraction as a factor reducing the amount of acrylamide in the product such as rusks by about 60% on average, the very important role of parameters such as the browning index or water activity as markers of the content of this carcinogen in the finished product was also indicated. The concentration of 15% of oat fiber preparations was considered the most optimal share in the recipe composition of rusks, regardless of the share of β-glucan fraction in each fiber preparation, i.e., 20 or 30%. At the same time, using this concentration, the obtained rusks were characterized by high sensory acceptability. This is one of the important factors that determine the choice of this product by a potential consumer. A clear benefit of the obtained research results for food producers is the indication of a way to significantly reduce acrylamide by approximately 70% in confectionery products. This will enable potential producers of these products to adapt to the current trend of striving to reduce the content of carcinogen AA, among others, in confectionery products, including dietary bread (rusks). Moreover, the use of B-glucans from a by-product of the milling industry, i.e., bran, to significantly reduce acrylamide in food processing is consistent with the assumptions of a circular industry. In further studies, other soluble fiber preparations should also be used to check whether they could be used effectively as a potential tool to significantly reduce acrylamide in food products, i.e., by at least 50%, compared to the control sample. Thus, a wide group of food products would have a chance to become safer for potential consumers, including people after severe convalescence. acrylamide content but would also have a chance to be enriched with soluble dietary fiber.

## Figures and Tables

**Table 1:** Free carbohydrate concentrations in the doughs before and after the fermentation process depending on the type and concentration of the oat fiber preparation used in the recipe composition.

	Blank Sample		10% Addition of OFP^20% β-glucans^		10% Addition of OFP^30% β-glucans^		15% Addition of OFP^20% β-glucans^		15% Addition of OFP^30% β-glucans^		20% Addition of OFP^20% β-glucans^		20% Addition of OFP^30% β-glucans^	
	(mg g ^−1^d.m.)													
	Fermentation		Fermentation		Fermentation		Fermentation		Fermentation		Fermentation		Fermentation	
	Before	After	Before	After	Before	After	Before	After	Before	After	Before	After	Before	After
Fructose	23.43 ^Aa^ ±0.12	22.21 ^Ba^ ±0.13	23.70 ^Ab^ ±0.10	22.51 ^Ba^ ±0.18	21.13 ^Ac^ ±0.10	20.98 ^Ab^ ±0.11	23.93 ^Ad^ ±0.12	23.45 ^Bc^ ±0.14	21.09 ^Ac^ ±0.13	20.18 ^Bd^ ±0.16	21.76 ^Ad^ ±0.13	21.01 ^Bb^ ±0.11	24.51 ^Ae^ ±0.13	23.04 ^Bf^ ±0.11
Glucose	15.14 ^Aa^ ±0.03	11.24 ^Ba^ ±0.14	14.92 ^Ab^ ±0.11	14.21 ^Bb^ ±0.11	13.74 ^Ac^ ±0.12	12.89 ^Bc^ ±0.10	14,51 ^Ad^ ±0.09	13.45 ^Bd^ ±0.16	13.31 ^Ae^ ±0.15	12.28 ^Be^ ±0.17	14.43 ^Ad^ ±0.09	13.68 ^Bd^ ±0.14	17.93 ^Af^ ±0.14	16.15 ^Bf^ ±0.11
Sucrose	0.61 ^Aa^ ±0.11	0.42 ^Aa^ ±0.11	0.47 ^Aa^ ±0.11	0.28 ^Aa^ ±0.11	0.70 ^Aa^ ±0.13	0.54 ^Aab^ ±0.13	0.76 ^Aa^ ±0.14	0.61 ^Ab^ ±0.11	0.13 ^Ab^ ±0.11	0.10 ^Ac^ ±0.15	0.14 ^Ab^ ±0.11	0.09 ^Ac^ ±0.07	0.10 ^Ab^ ±0.09	0.08 ^Ac^ ±0.06
Maltose	23.23 ^Aa^ ±0.04	16.67 ^Ba^ ±0.12	28.52 ^Ab^ ±0.14	21.25 ^Bb^ ±0.14	27.64 ^Ac^ ±0.11	21.05 ^Bb^ ±0.11	27.31 ^Ac^ ±0.15	20.27 ^Bc^ ±0.14	27.64 ^Ac^ ±0.12	21.06 ^Bb^ ±0.11	28.58 ^Ab^ ±0.16	22.45 ^Bd^ ±0.11	26.19 ^Ad^ ±0.14	21.89 ^Be^ ±0.17
Sum	61.41 ^Aa^ ±0.15	50.44 ^Ba^ ±0.17	67.61 ^Ab^ ±0.11	58.25 ^Bb^ ±0.10	63.21 ^Ac^ ±0.12	55.46 ^Bc^ ±0.09	66.51 ^Ad^ ±0.11	57.78 ^Bd^ ±0.13	62.17 ^Ae^ ±0.13	53.62 ^Be^ ±0.14	64.85 ^Af^ ±0.15	57.23 ^Bf^ ±0.14	68.73 ^Ag^ ±0.16	61.16 ^Bg^ ±0.14

Blank sample-dough without the addition of an oat fiber preparation; OFP^20% β-glucans^-oat fiber preparation with a 20% β-glucans content; OFP^30% β-glucans^-oat fiber preparation with a 30% β-glucans content; uppercase letters-different letters within the same row indicate significant differences in the content of a given type of carbohydrate depending on the fermentation process (before or after) for a given concentration of the fiber preparation (*n* = 3; *p* ≤ 0.05); lowercase letters-different letters within the same row indicate significant differences in the content of a given type of carbohydrate depending on the concentration of the added fiber preparation before or after the fermentation process (*n* = 3; *p* ≤ 0.05); data are presented as the mean ± SD.

**Table 2:** Free carbohydrate concentrations in the baked cakes and rusks depending on the type and concentration of the oat fiber preparation used in the recipe composition.

	Blank Sample		10% Addition of OFP^20% β-glucans^		10% Addition of OFP^30% β-glucans^		15% Addition of OFP^20% β-glucans^		15% Addition of OFP^30% β-glucans^		20% Addition of OFP^20% β-glucans^		20% Addition of OFP^30% β-glucans^	
	(mg g^−1^ d.m.)													
	Baked Cake	Rusk	Baked Cake	Rusk	Baked Cake	Rusk	Baked Cake	Rusk	Baked Cake	Rusk	Baked Cake	Rusk	Baked Cake	Rusk
Fructose	11.95 ^Aa^ ±0.12	5.45 ^Ba^ ±0.02	19.65 ^Ab^ ±0.11	14.68 ^Bb^ ±0.10	17.27 ^Ac^ ±0.15	15.05 ^Bc^ ±0.11	22.28 ^Ad^ ±0.11	17.19 ^Bd^ ±0.18	20.09 ^Ae^ ±0.10	16.89 ^Bd^ ±0.14	17.10 ^Ac^ ±0.10	16.48 ^Bde^ ±0.11	17.70 ^Af^ ±0.13	16.00 ^Bf^ ±0.11
Glucose	7.00 ^Aa^ ±0.03	2.54 ^Ba^ ±0.14	13.90 ^Ab^ ±0.12	10.21 ^Bb^ ±0.15	10.86 ^Ac^ ±0.14	9.89 ^Bc^ ±0.12	12.35 ^Ad^ ±0.16	11.45 ^Bd^ ±0.15	10.20 ^Ae^ ±0.11	9.28 ^Be^ ±0.13	10.89 ^Ac^ ±0.10	7.68 ^Bf^ ±0.12	8.78 ^Af^ ±0.15	6.15 ^Bg^ ±0.14
Sucrose	nd	nd	0.19 ^Aa^ ±0.10	0.08 ^Aa^ ±0.04	0.46 ^Ab^ ±0.04	0.24 ^Bb^ ±0.03	0.56 ^Ac^ ±0.04	0.31 ^Bd^ ±0.07	0.09 ^Ad^ ±0.02	0.04 ^Bc^ ±0.01	0.06 ^Ae^ ±0.01	0.02 ^Bd^ ±0.00	nd	nd
Maltose	1.15 ^Aa^ ±0.04	0.55 ^Ba^ ±0.02	8.52 ^Ab^ ±0.11	4.25 ^Bb^ ±0.13	7.44 ^Ac^ ±0.13	3.15 ^Bc^ ±0.10	6.89 ^Ad^ ±0.11	2.27 ^Bd^ ±0.11	7.04 ^Ac^ ±0.10	4.06 ^Bb^ ±0.16	8.86 ^Ae^ ±0.14	2.41 ^Bd^ ±0.09	6.18 ^Af^ ±0.11	1.89 ^Bf^ ±0.13
Sum	20.10 ^Aa^ ±0.15	8.54 ^Ba^ ±0.03	42.26 ^Ab^ ±0.11	29.22 ^Bb^ ±0.13	36.03 ^Ac^ ±0.11	28.33 ^Bc^ ±0.08	42.08 ^Ab^ ±0.14	31.22 ^Bd^ ±0.16	37.42 ^Ad^ ±0.11	30.27 ^Be^ ±0.15	36.91 ^Ad^ ±0.11	26.59 ^Bf^ ±0.10	32.66 ^Ae^ ±0.13	24.04 ^Bg^ ±0.13

Blank sample-dough without the addition of an oat fiber preparation; OFP^20% β-glucans^-oat fiber preparation with a 20% β-glucans content; OFP^30% β-glucans^-oat fiber preparation with a 30% β-glucans content; uppercase letters-different letters within the same row indicate significant differences in the content of a given carbohydrate depending on the type of product (baked cake or rusk) for a given concentration of the fiber preparation (*n* = 3; *p* ≤ 0.05); lowercase letters-different letters within the same row indicate significant differences in the content of a given carbohydrate depending on the concentration of the added fiber preparation for a given type of product (baked cake or rusk) (*n* = 3; *p* ≤ 0.05); data are presented as the mean ± SD.

**Table 3:** The effect of the type and concentration of the oat fiber preparation used in the recipe composition on the color parameters of baked cakes and rusks.

	Blank Sample		10% Addition of OFP^20% β-glucans^		10% Addition of OFP^30% β-glucans^		15% Addition of OFP^20% β-glucans^		15% Addition of OFP^30% β-glucans^		20% Addition of OFP^20% β-glucans^		20% Addition of OFP^30% β-glucans^	
	Baked Cake	Rusk	Baked Cake	Rusk	Baked Cake	Rusk	Baked Cake	Rusk	Baked Cake	Rusk	Baked Cake	Rusk	Baked Cake	Rusk
L*	71.92 ^Aa^ ±0.98	72.48 ^Aa^ ±0.46	72.11 ^Aa^ ±0.31	71.73 ^Bb^ ±0.23	73.46 ^Ab^ ±0.45	72.21 ^Bab^ ±0.54	75.90 ^Ac^ ±0.29	72.72 ^Bab^ ±0.34	77.60 ^Ad^ ±0.49	74.23 ^Bb^ ±1.02	73.37 ^Aba^ ±0.87	73.34 ^Ab^ ±0.19	71.93 ^Aa^ ±0.61	72.08 ^Aab^ ±0.58
a*	2.73 ^Aa^ ±0.63	2.81 ^Aa^ ±0.34	2.92 ^Aa^ ±0.11	3.48 ^Bb^ ±0.15	2.59 ^Aa^ ±0.19	2.48 ^Aa^ ±0.72	1.19 ^Ab^ ±0.26	3.14 ^Bba^ ±0.22	1.74 ^Ab^ ±0.34	1.96 ^Ac^ ±0.28	1.89 ^Ab^ ±0.27	2.69 ^Ba^ ±0.18	2.79 ^Aa^ ±0.31	2.88 ^Aa^ ±0.32
b*	22.48 ^Aa^ ±0.44	26.31 ^Ba^ ±0.56	21.31 ^Aa^ ±0.66	22.11 ^Ab^ ±0.57	19.19 ^Aa^ ±0.43	21.41 ^Bc^ ±0.12	17.17 ^Ab^ ±0.32	16.54 ^Bd^ ±0.23	16.98 ^Ab^ ±0.24	18.41 ^Be^ ±0.33	20.63 ^Ac^ ±0.21	18.91 ^Be^ ±0.98	20.93 ^Ac^ ±0.31	22.21 ^Bb^ ±0.34
ΔE	standard	standard	1.20 ^Aa^ ±0.42	4.29 ^Ba^ ±0.31	3.63 ^Ab^ ±0.28	4.92 ^Ba^ ±0.43	6.81 ^Ac^ ±0.98	9.78 ^Bb^ ±0.71	7.97 ^Ac^ ±0.41	8.14 ^Ac^ ±0.28	2.50 ^Aab^ ±0.94	7.45 ^Bc^ ±0.76	1.55 ^Aab^ ±0.41	4.12 ^Ba^ ±0.28
Brightness (D65)	standard	standard	0.19 brighter	0.75 darker	1.54 brighter	0.27 darker	3.98 brighter	0.24 brighter	5.68 brighter	1.75 brighter	1.45 brighter	0.86 brighter	0.01 brighter	0.40 darker
BI	39.47 ^Aa^ ±0.14	46.88 ^Ba^ ±0.98	37.29 ^Ab^ ±0.13	39.65 ^Bb^ ±0.28	32.23 ^Ac^ ±0.67	36.94 ^Bc^ ±0.78	26.23 ^Ad^ ±0.11	28.47 ^Bd^ ±0.96	25.82 ^Ae^ ±0.10	29.82 ^Bd^ ±0.16	31.06 ^Af^ ±0.11	31.94 ^Ad^ ±0.98	31.76 ^Af^ ±1.02	39.00 ^Be^ ±0.97

Blank sample-dough without the addition of an oat fiber preparation; OFP^20% β-glucans^-oat fiber preparation with a 20% β-glucans content; OFP^30% β-glucans^-oat fiber preparation with a 30% β-glucans content; L*-lightness; a*-redness/greenness; b*-yellowness/blueness; ΔE—total color difference; BI-browning index; uppercase letters-different letters within the same row indicate significant differences in the value of a given parameter depending on the type of product (baked cake or rusk) for a given concentration of the fiber preparation (n = 3; *p* ≤ 0.05); lowercase letters-different letters within the same row indicate significant differences in the value of a given parameter depending on the concentration of the added fiber preparation for a given type of product (baked cake or rusk) (*n* = 3; *p* ≤ 0.05); data are presented as the mean ± SD.

## Data Availability

Data are available upon request because of restrictions, e.g., privacy or ethical reasons.
